# Etiological risk factors for subfertility among Palestinian women in Gaza

**DOI:** 10.7555/JBR.27.20120104

**Published:** 2013-01-30

**Authors:** Mahmoud Mohammed Sirdah, Abdelnasser Kassem Abushahla, Bahaa Yousif Ghalayeni, Ahmed Gamel Aburamadan

**Affiliations:** a Biology Department, Al Azhar University Gaza, Palestine;; b Alawda Maternity Hospital, Gaza, Palestine;; c Al Basma Fertility Center, Gaza, Palestine.

**Keywords:** subfertility, etiology, risk factors, Gaza, Palestine

## Abstract

The inability to procreate is frequently considered a personal tragedy and a hardship for couples, impacting on the entire family and even the local community. In Gaza strip, Palestine, there has been no study on etiological risk factors for subfertility. The present study aimed to identify risk factors associated with subfertility among women in Gaza, Palestine. One hundred and sixty-nine women in the study group and 115 women in the control group were included. Cases were selected randomly from those referred to the Al Basma Fertility Center, Gaza, Palestine. Data were collected through close-ended questionnaire, sonography, hormonal analysis and thrombophilia profile that included *the methylenetetrahydrofolate redu*ctase (*MTHFR* 677 C > T), *factor V leiden* (1691 G > A) and *prothromb*in (20210 G > A) genes. By using univariate analyses, the effects of different patient-related variables on the presence of subfertility were evaluated. A multiple logistic regression model was constructed, crude and adjusted odds ratios (OR) and 95% confidence intervals (95% CI) were calculated. The findings showed that 73.5 % (169/230) of the women referred to the Al Basma Center sought treatment for subfertility. Different etiological risk factors were associated with subfertility, the most frequent of which in descending order were: thrombophilic disorders, fallopian tube problems, sex hormone abnormalities and polycystic ovary syndrome with an adjusted OR of 21.42, 13.63, 11.69 and 10.29, respectively. In conclusion, several etiological risk factors are responsible for subfertility among women in Gaza. Comprehensive evaluation of infertile women should be considered in the course of treatment; otherwise, the duration of sterility may be extended.

## INTRODUCTION

Conception and pregnancy are complex processes that include different biological aspects and phases. The most important things are the production of healthy sperms and eggs from the husband and wife's reproductive organs. The inability to conceive after at least one year of regular unprotected sexual intercourse can be due to different causes related to either partner or to both. In developing countries and especially in the Middle East, a high rate of childlessness is considered as one of the most important and underappreciated reproductive health problems[1],[2]. The inability to procreate is considered a personal tragedy with considerable suffering for both partners, affecting the entire family and even local community, which may lead to severe psychosocial consequences[3],[4]. In many cultures, including the Arabic oriental, womanhood is defined through motherhood, and infertile women usually carry, incorrectly, the responsibility for couple's inability to conceive or having a baby. Childless women are, unfortunately, commonly stigmatized, resulting in isolation, disregard and disrespect[5]–[8].

The World Health Organization (WHO) estimates that there were 60-80 million infertile couples worldwide with an incidence of 20% in the Eastern Mediterranean region and 11% in the developed world[9],[10]. In Palestine, there have been no documented reports or published scientific works that investigated the magnitude and etiological risk factors associated with subfertility. Moreover, the demographic records published by the Palestinian Central Bureau of Statistics revealed a significant decline in fertility rates of Palestine. The fertility rate declined from 6.1 births per woman in 1994 to 4.9 in 1999 and 4.6 in 2003 to 4.1 in 2008-2009[11]. As the considerably high prevalence of childlessness worldwide and especially in developing countries, we believed that the identification of etiological risk factors underlying each case is the key to successful treatment and problem solving for so many cases. The present work aimed for indentifying the etiological risk factors associated with subfertility among Palestinian women in Gaza.

## SUBJECTS AND METHODS

### Subjects

The present study took place in Gaza and the mentioned data represent cases from January 2006 to May 2010. The cases were selected randomly (simple random sampling) from 169 infertile women who attended the Al Basma Fertility Center for medical management of subfertility or other maternity issues. The Al Basma Fertility Center is located in Gaza city and offers its service to couples from all Gaza Strip localities. In addition, 115 healthy fertile women who attended public or private clinics for family planning issues were included as the control group. Women who received any medication or were subjected to surgery to achieve pregnancy were excluded from the control group. The study was performed in accordance with the ethical standards established in the 1964 and 1975 Declarations of Helsinki, and the modifications thereafter. The present study was approved and endorsed by the Ethical Committee at the Biology Department, Al Azhar University-Gaza. Informed consent was obtained from all women prior to their inclusion in the study.

### Questionnaire interview

Parts of data were collected by using close-ended questionnaire which was constructed and conducted in Arabic language. The questionnaire was designed to include major components: socio-demographic and general characteristics; gynecological and obstetrics profile; other health characteristics; health complains and medical history of the subjects. The items and components of the questionnaire were validated at three levels including the criterion, content and piloting.

### Ultrasonography

After the completion of questionnaire, women were clinically investigated by gynecologists for the status of the reproductive system using ultrasound-based diagnostic imaging technique. Ultrasonography was conducted by using an Aloka SSD-1000 Ultrasound (Aloka, Tokyo, Japan). Ultrasonography provided an evaluation of the anatomical characteristics of the female reproductive system. The main points of interest were the size of the uterus, presence of myoma, the state of the endometrium, polycystic ovary syndrome, the presence of hydrosalpinges and cysts or ovarian neoplasm.

### Infections

Gynecologists also evaluated the women for different causes of vaginal infections using high vaginal swabs, which are helpful for the diagnosis of bacterial vaginosis and vaginal candidiasis. The high vaginal swab technique included a sterile swab that was inserted into the vaginal posterior fornix while care was taken to avoid the lower vaginal walls[12]. The swabs were used for identifying the causative microorganisms of vaginal infection by using culture technique. In addition, fresh mid-urine specimens were investigated microscopically for microbiological infections. For infection with *Toxoplasma gondii*, venous blood samples were withdrawn and serum was tested for specific IgM antibodies by using ELISA test kit (Teco Diagnostics, Anaheim, CA, USA) following the manufacturer's instructions.

### Hormonal analysis

By using commercially available kits (Tosoh Bioscience, San Francisco, CA, USA) and according to the manufacturer's instructions, venous blood from the subjects was tested for the following hormones, including prolactin, follicle stimulating hormone (FSH), luteinizing hormone (LH), thyroid stimulating hormone (TSH) and anti-mullerian hormone (AMH). Any change from the normal ranges provided in the instruction manual of Tosoh Biosciences kits was considered as hormonal abnormality.

### Thrombophilic profile

In addition, the women were investigated for heterozygosity or homozygosity in one or more of the three common genes affecting the coagulation factors in the clotting cascades. The polymorphism was investigated by PCR/RFLP. The investigated gene variants were *MTHFR* 677 C > T and digested by Hinf I, *factor V Leiden* 1691 G > A digested by *Mnl* I and prothrombin 20210 G > A digested by *Hind* III. The molecular methodologies were as described elsewhere[13]–[15]. For cost-effectiveness purpose, it was worthwhile to mention that, in the control group, we only screened 35 randomly selected samples for the polymorphism in the included thrombophilic genes.

### Statistical analysis

The frequencies of different categorical variables among subfertile women and fertile women in the control group were summarized as numbers and frequencies and compared by using χ^2^ tests. The association between subfertility and variables were assessed using a univariate simple logistic regression and reported as crude odds ratios (OR) with 95% confidence intervals (95% CI). Statistically significant predictor variables with *P* value less than 0.05 in the univariate analysis were included in the multiple logistic regression model to examine the risk factors that were potentially associated with subfertility in our Palestinian cohort. The results from the multiple logistic regression were presented as adjusted odds ratio (aORs) with 95% CI. *P* values were two sided, and *P* < 0.05 was considered to indicate statistical significance. All analyses were performed by using the SPSS statistical package version 13.0 (SPSS Inc, Chicago, IL, USA).

## RESULTS

The results showed that 73.5 % (169/230) of the women who attended gynecology clinics sought treatment for subfertility and 26.5% (61/230) sought treatment for recurrent miscarriages. The mean age of subfertile women was 27.5±5.7 years, and 75.1 % of them were ≤ 30 years old. Statistically significant differences were noted in the prevalence of subfertility between different localities of Gaza. The percentage of subfertility in urban areas was significantly higher than that of rural areas (*P* < 0.001) (***Table 1***). Moreover, the results revealed significant differences with regard to career (*P* = 0.025): the percentage of subfertility among career women was significantly higher than that of non-career women while no significant differences (*P* = 0.504) were reported with regard to educational level.

**Table 1:** Socio-demographic characteristics of the subjects

Variables	Normal group [*n*=115, *n*(%)]	Subfertility group [*n*=169, *n*(%)]		*P*-value*
Locality				
Urban	96(83.4)	165(97.6)		< 0.001
Rural	19(16.6)	4(2.4)		
Employment				
Working	10(8.7)0	31(18.3)		0.025
Not working	105(91.3) 0	138(81.7)		
Education				
School	80(69.6)	124(73.4)		0.504
Graduate	35(30.4)	45(26.6)		

*P values were derived from the χ^2^ test of association between groups.

The evaluation of menstrual cycle and the female reproductive system is illustrated in ***Table 2***. Significantly higher percentages of women with irregular menstrual cycle and period < 5 days were reported in the subfertile group (10.1% and 12.4%) as compared to the control group (3.5% and 5.2%), with *P* values of 0.04 and 0.048, respectively, while no significant differences were reported for the severity/heaviness of menstruation (*P* = 0.083).

**Table 2:** Characteristics of menstruation and the female reproductive system of the study subjects

Characteristics	Normal group [*n* =115, *n*(%)]	Subfertility group [*n* =169, *n*(%)]	*P*-value*
Regularity of menstruation			
Irregular	4(3.5)	17(10.1)	0.04
Length of menstruation			
< 5 days	6(5.2)	21(12.4)	0.048
5-6 days	95(82.6)	121(71.6)0	
> 6 days	14(12.2)	27(16.0)	
Amount of menstruation			
Heavy	11(9.6)0	7(4.1)	0.083
Retroverted uterus			
Yes	3(2.6)0	31(18.3)	< 0.001
Polycystic ovary syndrome			
Yes	2(1.7)	30(17.8)	< 0.001
Abnormalities of fallopian tube			
Yes	1(0.9)	22(13.0)	< 0.001
Endometriosis			
Yes	1(0.9)	7(4.1)	0.148
Presence of uterus myoma			
Yes	0(0)0	12(7.1)0	N.A

*P values were derived from the χ^2^ test of association between groups, N.A: not applicable.

In addition, the percentage of subfertile women (18.3%) with retroverted uterus was significantly higher than that in the control group (2.6%) (*P* < 0.0001). The percentages of women with polycystic ovary syndrome and abnormities in fallopian tubes were also significantly higher in the subfertility group than those of the control group (*P* < 0.001). On the other hand, no significant differences were noted in the prevalence of endometriosis between the subfertile and control group (*P* = 0.148).

The classification of the study population according to the presence of abnormalities in one or more of fertility-related hormones (sex hormones and TSH) are presented in ***Table 3***. Hormonal abnormities were significantly higher in the subfertile group as compared to the control group. In addition, the percentages of women with a family history of subfertility, women with vaginal infections and women with chlamydia were significantly higher in subfertile women as compared to the control group while no significant differences were observed regarding urinary tract infections or toxoplasmosis (*P* > 0.05).

**Table 3:** Medical characteristics of the subjects

Characteristics	Normal group [*n*=115, *n*(%)]	Subfertility group [*n*=169, *n*(%)]	*P*-value*	
Sex hormone abnormalities				
Yes	5(4.3)	80(47.3)	< 0.001	
TSH abnormalities				
Yes	1(0.9)	13(7.7)0	0.01	
Family history of subfertility				
Yes	2(1.7)	14(8.3)0	0.019	
Vaginal infections				
Yes	18(15.7)	76(45.0)	< 0.001	
Vaginal infections				
Yes	18(15.7)	76(45.0)	< 0.001	
Urinary tract infections				
Yes	8(7.0)	21(12.4)	0.164	
Chlamydia				
Yes	1(0.9)	12(7.1)	0.018	
Toxoplasmosis				
Yes	1(0.9)	8(4.7)	0.089	

*P values were derived from the χ^2^ test of association between groups.

***Table 4*** shows the distributions of some common thrombophilic abnormalities among the study population, which were also shown as an overall prevalence of thrombophilic disorders for the cases and controls. The overall prevalence of thrombophilic disorders was not a mathematical sum of the prevalence of individual polymorphisms, as one sample may contain one or more polymorphisms. About 30.2% of the women in the subfertile group exhibited one or more of the thrombophilic disorders screened. These percentages were significantly higher when compared to those of the control group (*P* = 0.022). The detailed distributions of the thrombophilic disorders among the cases are also presented.

**Table 4:** Prevalence of thrombophilic disorders among the subjects

Type of thrombophilic disorders	Normal group [*n* = 35^€^, *n*(%)]	Subfertility group [*n* = 169, *n*(%)]	*P*-value*
Methylenetetrahydrofolate reductase (MTHFR)			
Normal	33(94.3)	127(75.1)	0.012
Homozygous/heterozygous abnormal gene	2(5.7)	42(24.9)	
Factor V Leiden			
Normal	32(91.4)	157(92.9)	0.726
Homozygous/heterozygous abnormal gene	3(8.6)	12(7.1)	
Prothrombin			
Normal	34(97.1)	163(96.4)	1.00
Homozygous/heterozygous abnormal gene	1(2.9)	6(3.6)	
Overall thrombophilic disorder	4(11.4)	51(30.2*)	0.022

*P values were derived from the χ^2^ test of association between groups. € The 35 samples represent the whole controls as they were randomly selected from the 115 controls.

We performed univariate statistical analysis to study the association between the various exposures, if any, with the fertility state (subfertility versus fertile controls). In this unadjusted analysis, we calculated the crude ORs and their 95% CI, which are shown in ***Table 5***. In this analysis, the following factors: women's educational level, heaviness of menstruation, endometriosis, urinary tract infection, and toxoplasmosis showed no significant associations. As a result, our multivariate analysis was adjusted to contain fewer potential risk factors. After adjustment of the potential confounding factors as a result of the univariate analysis, we undertook a multivariate statistical analysis in order to calculate the aOR and their 95% CI aiming to identify the etiological risk factors associated to subfertility in our cohort of Palestinian women. The multiple logistic regression analysis for all cases and controls provided nearly similar results (***Table 6***) to the unadjusted analyses except for three factors, including family history, irregularity of menstruation and thyroid stimulating hormone abnormalities.

**Table 5:** Univariate analysis: crude ORs and their 95% CI

Variable	Crude ORs	95% CI
Locality		
Rural	1.00	--
Urban	8.16	2.70-24.70
Working status		
Housewife	1.00	--
Working	2.36	1.12-5.03
Education		
Graduate	1.00	--
School	1.21	0.71-2.04
Family history of subfertility	5.10	1.14-22.9
Irregularity of menstruation	0.32	0.11-0.98
Length of menstruation		
5 days or more	1.00	--
< 5 days	2.58	1.01-6.60
Heavy menstruation	2.45	0.92-6.52
Retroverted uterus	8.39	2.50-28.15
Polycystic ovary syndrome	12.19	2.85-52.13
Abnormalities of the fallopian tube	20.73	2.77-155.06
Endometriosis	4.93	0.6-40.59
Sex hormone abnormalities	19.78	7.68-50.92
TSH abnormalities	9.50	1.23-73.67
Vaginal infections	4.40	2.45-7.92
Urinary tract infections	1.90	0.81-4.45
Chlamydia	8.71	1.12-67.97
Toxoplasmosis	5.67	0.70-45.92
Thrombophilic disorders	3.35	1.12-9.98

**Table 6:** Multivariate analysis: aOR and their 95% CI for risk factors associated with subfertility

Variable	aOR	95% CI	P-value
Locality			
Rural	1.00	--	
Urban	6.97	2.29-21.21	0.001
Working women			
Housewife	1.00	--	
Working	1.92	0.89-4.150	0.090
Family history of subfertility	4.17	0.92-18.86	0.640
Irregularity of menstruation	0.38	0.09-1.760	0.220
Length of menstruation			
5 days or more	1.00	--	
< 5 days	3.80	1.04-13.92	0.043
Retroverted uterus	5.52	1.21-25.05	0.027
Polycystic ovary syndrome	10.29	1.88-56.10	0.007
Abnormalities of fallopian tube	13.63	01.43-129.81	0.023
Sex hormones abnormalities	11.69	3.82-35.80	0.001
TSH abnormalities	1.43	0.14-14.33	0.760
Vaginal infections	3.56	1.57-8.06	0.002
Thrombophilic disorder	21.42	6.80-67.51	0.001

aOR: adjusted odds ratio.

## DISCUSSION

This is the first study that attempts to highlight possible etiological risk factors among subfertile Palestinian women in Gaza where Arabic and oriental traditions and customs control the attitudes of society. In this study, we aimed not only to present the frequencies of factors that may possibly cause subfertility, but also to provide a more useful elucidation of the attributable risks in terms of the whole population from the public health perspective. Therefore, we used the univariate and multivariate statistical analyses and provided the crude and adjusted OR and their 95% CI, which allow health epidemiologists to calculate what percentage of a disease could possibly be prevented if an etiological risk factor could be removed or decreased from the population.

The most frequent etiological risk factors for subfertility among Palestinian women in Gaza were thrombophilic disorders (aOR = 21.42, 95% CI, 6.80-67.51, *P* = 0.001), fallopian tube problems (aOR = 13.63, 95% CI, 1.43-129.81, *P* = 0.023), sex hormones abnormalities (aOR = 11; 95% CI, 69 3.82-35.80, *P* = 0.001) and polycystic ovary syndrome (aOR = 10.29, 95% CI, 1.88-56.10, *P* = 0.007). When these etiological risk factors were discussed in terms of aOR, in addition to prevalence, the magnitude of these factors became more disquieting and frightening. Women with thrombophilic disorders were about 21 times more likely to be subfertile than those with normal thrombophilic profile. The same is true for fallopian tube damage, sex hormones abnormalities and polycystic ovary syndrome.

The present study investigated the thrombophilic profile of subfertile women and the results highlighted the importance of these disorders in developing subfertility in Gaza women. In our cohort, thrombophilic disorders ranked first as a major etiological factor for subfertility among Palestinian women at the Gaza Strip. The results revealed that women with thrombophilic disorders are about 21 times more likely to be subfertile than those with normal thrombophilic profile. Therefore, testing and managing these thrombophilic disorders in women before conception are highly recommended. The crucial effects of thrombophilic disorders on conception and pregnancy outcome have been addressed properly in previous investigations[16]–[21]. Fatini et al. demonstrated that a significant relationship between inherited thrombophilia and unexplained infertility in 930 infertile females of Caucasian origin, thus suggesting the contribution of genetic components in modulating unexplained infertility[22]. Thus, several subfertile women cases in Gaza could be therapeutically managed by correcting or adjusting their coagulation profile before and during the childbearing period. However, Casadei et al. found no significant difference in the prevalence of three thrombophilic mutations (MTHFR 677 C > T; Factor V Leiden 1691 G > A and prothrombin 20210 G > A) between 100 infertile women with unexplained infertility as compared with 200 control fertile women without a history of infertility[23].

Fallopian tube problems and sex hormone abnormalities ranked second and third as major etiological risk factors for subfertility among Gaza women, respectively. Thus, several subfertile women cases in Gaza could be managed before the planning of childbearing. Studies addressing fallopian tube problems, sex hormone abnormalities and polycystic ovary syndrome as major risk factors for subfertility in other populations and settings are abundant[24]–[30]. Some of the risk factors addressed by the present work have been documented in previous studies. In our study, retroverted uterus and vaginal infections are among the etiological risk factors of subfertility, while infection with chlamydia, the presence of toxoplasmosis and urinary tract infections are not among significant risk factors in our cohort. The published data about the association between urogenital infections and female subfertility are conflicting. A study of 622 infertile and 495 fertile women failed to detect a relationship between toxoplasmosis and female subfertility[31], while the study by Li et al. of 882 subfertile and 107 fertile women revealed a significant difference in the seroprevalence of toxoplasmosis antibodies between subfertile (15.9%) and fertile women (5.6%)[32]. The negative impact of genital chlamydia trachomatis infections on female fertility has been well documented in previous studies and reviews[33]–[35].

El Hakim et al.[36] showed a direct association between chlamydia antibody titer and the severity of tubal damage in subfertile women. Cytomegalovirus infection has been associated wtih female subfertility[37]; however, the study by Mahdi et al. failed to reveal a significant difference between the infertile and control group regarding cytomegalovirus infection [38].

Moreover, hormonal abnormalities and urban life style were considered as risk factors in our study. Damti et al. hypothesized that alternation of cortisol excretion patterns along the menstrual cycle due to stress eventually affects women's hormonal profile in the critical stages of child bearing age[25]. In our study, many subfertile women were found to have a combination of different etiological factors. Therefore, it is the responsibility of gynecologists to consider the etiological risk factors mentioned in the present work during their evaluation of patients. Finding one risk factor, even a major risk factor, does not by any means restrict the seeking for other risk factors.

It is concluded that subfertility among Palestinian women of Gaza is a multi-factorial condition where several possible etiological factors could be recognized. High risk and lower risk factors were reported and should be considered during managing subfertile women for hopeful conception. Therefore, comprehensive evaluation for women should be a strategic approach; otherwise, waiting for motherhood will be longer or conception may never take place.
